# Epigallocatechin Gallate Enhances MAL-PDT Cytotoxic Effect on PDT-Resistant Skin Cancer Squamous Cells

**DOI:** 10.3390/ijms21093327

**Published:** 2020-05-08

**Authors:** Daniela León, Kurt Buchegger, Ramón Silva, Ismael Riquelme, Tamara Viscarra, Bárbara Mora-Lagos, Louise Zanella, Fabiola Schafer, Cristina Kurachi, Juan Carlos Roa, Carmen Ili, Priscilla Brebi

**Affiliations:** 1 Laboratory of Integrative Biology, Centro de Excelencia en Medicina Traslacional—Scientific and Technological Bioresource Nucleus (CEMT-BIOREN), Universidad de La Frontera, Temuco 4810296, Chile; dleon.garrido@gmail.com (D.L.); kurt.buchegger@ufrontera.cl (K.B.); tviscarra.alvarez@gmail.com (T.V.); barbara.moralagos@gmail.com (B.M.-L.); zanella.bio@gmail.com (L.Z.); 2 Department of Basic Sciences, School of Medicine, Universidad de La Frontera, Temuco 4811230, Chile; 3 Instituto de Ciencias Biomédicas, Facultad de Ciencias de la Salud. Universidad Autónoma de Chile, Temuco 4810101, Chile; rsilpez@gmail.com (R.S.); ismael.riquelme.contreras@gmail.com (I.R.); 4 Department of Medical Specialties, School of Medicine, Universidad de La Frontera, Temuco 4811230, Chile; fdschafe@gmail.com; 5 São Carlos Institute of Physics, University of São Paulo (USP), P.O. Box 369, São Carlos 13560-970, São Paulo, Brazil; cristina@ifsc.usp.br; 6 Department of Pathology, Pontificia Universidad Católica de Chile, Santiago 8330024, Chile; jcroa@med.puc.cl

**Keywords:** non-melanoma skin cancer, photodynamic therapy, squamous cell carcinoma, methyl aminolevulinate

## Abstract

Photodynamic therapy (PDT) has been used to treat certain types of non-melanoma skin cancer with promising results. However, some skin lesions have not fully responded to this treatment, suggesting a potential PDT-resistant phenotype. Therefore, novel therapeutic alternatives must be identified that improve PDT in resistant skin cancer. In this study, we analyzed the cell viability, intracellular protoporphyrin IX (PpIX) content and subcellular localization, proliferation profile, cell death, reactive oxygen species (ROS) detection and relative gene expression in PDT-resistant HSC-1 cells. PDT-resistant HSC-1 cells show a low quantity of protoporphyrin IX and low levels of ROS, and thus a low rate of death cell. Furthermore, the resistant phenotype showed a downregulation of *HSPB1*, *SLC15A2, FECH, SOD2* and an upregulation of *HMBS* and *BIRC5* genes. On the other hand, epigallocatechin gallate catechin enhanced the MAL-PDT effect, increasing levels of protoporphyrin IX and ROS, and killing 100% of resistant cells. The resistant MAL-PDT model of skin cancer squamous cells (HSC-1) is a reliable and useful tool to understand PDT cytotoxicity and cellular response. These resistant cells were successfully sensitized with epigallocatechin gallate catechin. The in vitro epigallocatechin gallate catechin effect as an enhancer of MAL-PDT in resistant cells is promising in the treatment of difficult skin cancer lesions.

## 1. Introduction

Non-melanoma skin cancer (NMSC) is the most frequent neoplasia worldwide [1]. Basal cell carcinomas comprise about 80% of NMSC. The remaining 20% correspond to squamous cell carcinomas (SCC) [2], which may be preceded by actinic keratosis (AK) or Bowen’s disease (BD) [3]. Conventional treatment methods for SCC, AK and BD include surgery and non-surgical therapies such as curettage, electrodessication and cryotherapy [4,5]. In addition, AK and BD can be treated with photodynamic therapy (PDT) [6].

PDT is a treatment that combines the use of light, a photosensitizer (PS), and oxygen of the cells. Briefly, the mechanism of PDT action starts when the PS enters and accumulates into cell, to then be excited by light at a specific wavelength [7]. Excited PS transfers energy to cellular oxygen, producing reactive oxygen species (ROS), mainly singlet oxygen (^1^O_2_) [8]. The ROS triggers cell death by apoptosis [9], autophagy, and/or necrosis [7] selectively in those cells which incorporated the PS molecules [10].

Many photosensitizers used in dermatological lesions are porphyrin derivatives, including prodrugs such as 5-aminolevulinic acid (5-ALA) and its methylated form, methyl aminolevulinate (MAL) [11]. Both compounds are precursors of protoporphyrin IX (PpIX), which is synthesized into cells via the heme group biosynthesis pathway and have the capacity to act as PS [11].

PDT has been widely used to treat preneoplastic and neoplastic skin lesions due to its minimal invasiveness that allows easy access to both extensive lesions and those in difficult locations, offering optimal cosmetic outcomes [12,13]. However, in the case of NMSC, the complete response rates of PDT varies between 37% and 100%, which could be explained by the type of lesion and follow-up time reported in each clinical trial [14,15,16,17,18,19]. Unfortunately, recurrence rates show an increase 6 or 12 months after PDT [14,16,18,20].

This recurrence could be associated with cell resistance to PDT, however there are no reports on resistance to this therapy as there is in radiation therapy or chemotherapy in the treatment of other cancers [21]. Despite this lack of information, several studies have tried to clarify the mechanisms involved in PDT resistance, mainly using cancer cell lines [22,23,24,25].

Some PDT resistance factors include the amount of oxygen (O_2_) present in the cell, the amount of PS taken up by the cell and its accumulation level, and the participation of molecules with antioxidant and prosurvival capacity [21,26]. For example, molecules involved in the synthesis and accumulation of PpIX include HMBS and ferrochelatase (FECH) enzymes, while transporters associated with efflux of PpIX from the cell include PEPT1, PEPT2, ABCG2, and ABCB6 [27,28,29,30]. Furthermore, antioxidant enzymes such as SOD1, SOD2, GPX1, and GSR could have a role in neutralizing ROS. In the same way, according to previous studies, proteins as HIF-1α, HSP27, HSP70, HSP90 and survivin stand out [31,32,33,34,35,36,37]. Therefore, the dysregulation of these molecules would support the resistance associated with PDT, becoming potential targets.

On the other hand, natural compounds, such as those derived from green tea, have been studied in depth because of their anti-cancer and preventive potential of different pathologies [38,39,40,41]. Thus, epigallocatechin gallate catechin (EGCG), the most abundant component in green tea with potent antioxidant activity, is important in the treatment and prevention of different types of cancer, especially gastrointestinal cancer [42].

EGCG is a molecule of interest because it has been shown to have an effect on cell signaling pathways associated with the regulation of growth pathways and apoptosis, for example by activating p53, p21, JNK 1/2 and inhibiting AKT, ERK1/2 [39,43,44].

Despite being a mainly antioxidant agent, it has also shown a pro-oxidant effect, which is associated with its structure. The EGCG molecule has three aromatic rings (A, B and D), which are linked to a pyran ring (C) [43,45]. In addition, previous studies have shown that EGCG improves the effect of PDT, both in vivo and in vitro as a treatment for human Jurkat cells (leukemia), TC-1 cells (mouse lung tumor), and BA cells (mouse breast cancer) [46,47,48].

Therefore, the aim of this study was to characterize a cell model (HSC-1 cells) resistant to MAL-PDT and evaluate the in vitro effect of epigallocatechin gallate (EGCG) as a PDT enhancer on this model.

## 2. Results

### 2.1. Generation of MAL-PDT Resistance in HSC-1 Cells

**Figure 1:**
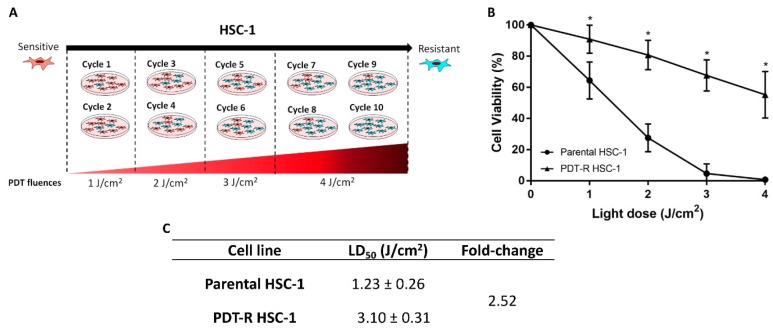
Generation of photodynamic therapy (PDT) resistance in HSC-1 cells. (**A**) The figure represents the workflow used to obtain a resistant population after 10 cycles of PDT. (**B**) Cell viability of parental HSC-1 cells compared to PDT-resistant HSC-1 cells at different light doses. (**C**) Lethal dose and fold-change index calculated for each cell line. Values of *p* < 0.05 were considered statistically significant. LD = Lethal dose; * *p* < 0.05. Data were expressed as mean ± SD of three biological replicates.

PDT resistance was developed by exposing HSC-1 cells to 10 cycles of increasing PDT fluences using a constant concentration of MAL (2 mM), as described by Milla et al. 2011 [35] (Figure 1A). Cell viability was evaluated in parental and resistant cell populations to PDT using MAL with different light fluences. We found that cell viability was significantly higher in PDT-resistant HSC-1 cells than parental cells at different light fluences. In this regard, light fluence at 4 J/cm^2^ can eliminate 100% of parental HSC-1 cells, whereas in PDT-resistant HSC-1 cells approximately 45% of the cell population was eliminated (Figure 1B). The fold-change index of PDT-resistant HSC-1 cells is 2.52-fold higher than parental cells (Figure 1C). These results are indicative of the emergence of a resistant phenotype in the PDT-resistant HSC-1 relative to its parent.

### 2.2. Characterization of MAL-PDT-Resistant Phenotype in HSC-1 Cells

In order to characterize the resistant cells according to cell death and proliferation process, we performed phosphatidylserine (PS) translocation (early event of apoptosis), cell death (late event of apoptosis), as well as clonogenic and wound healing assays. 

**Figure 2:**
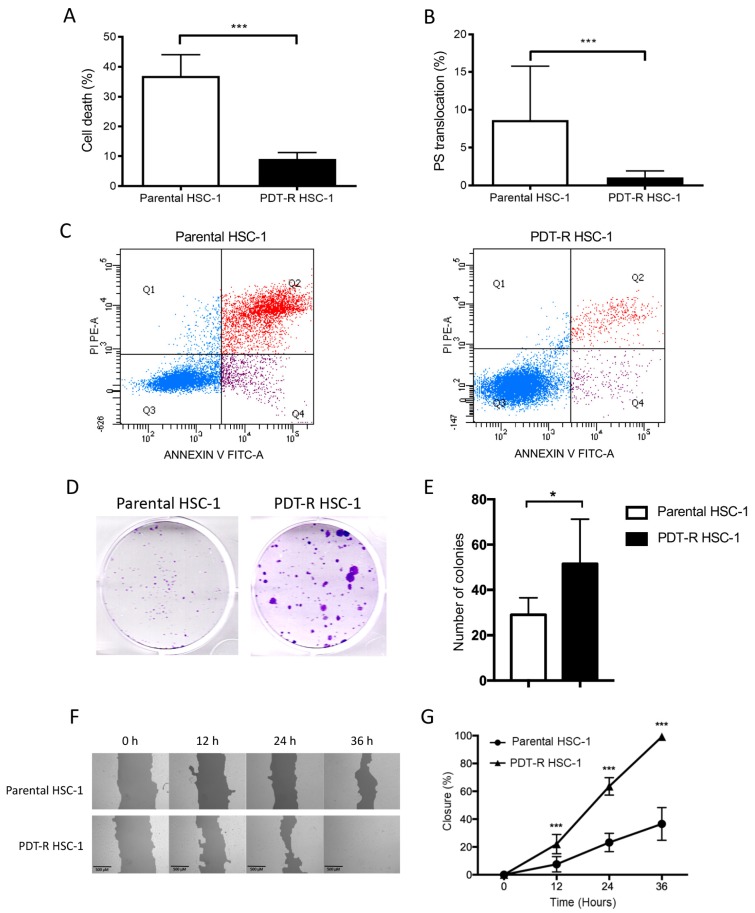
Cell death and proliferation capacity analysis. (**A**) Cell death analysis (AV (+) and PI (+)); (**B**) PS translocation (AV (+)). (**C**) Representative plot of the flow cytometry for cell death and PS translocation assays in parent and resistant cells. (**D**) Clonogenic assay, 500 cells were seeded in each plate for 14 days. (**E**) Quantification of colonies formed in parental HSC-1 and resistant cells. (**F**) Representative images of wound closure in parental HSC-1 and resistant cells. (**G**) Percentage of wound closure in resistant cells compared to parental HSC-1. Values of *p* < 0.05 were considered statistically significant. * *p* < 0.05; *** *p* < 0.001. Data were expressed as mean ± SD of three biological replicates.

After one-hour post-treatment with MAL-PDT (4 J/cm^2^), parental HSC-1 cells showed a significant increase in PS translocation (8.63 ± 7.13%) and cell death (36.96 ± 7.08%) compared to PDT-resistant HSC-1 cells (1.06 ± 0.87% and 9.04 ± 2.21%, respectively) (Figure 2A,B, *p* < 0.0005). Representative cytometric profiles according to cell death (Q1, Q2, and Q4) and PS translocation (Q4) are shown in Figure 2C.

According to cell proliferation capacity, we evaluated the ability to form colonies and wound healing. First, the colonies formed in both parental and PDT-resistant HSC-1 cells. PDT-resistant HSC-1 cells formed a higher number of colonies and larger than parental HSC-1 cells (Figure 2D,E). The wound healing assay showed that parental HSC-1 cells had 35.57 ± 11.78% wound closure, while PDT-resistant HSC-1 cells closed the entire area at 36 h post-scratch (Figure 2F,G).

These results show that resistant HSC-1 cells are capable of avoiding cell death and proliferating at a higher rate than parent cells.

### 2.3. PDT-Resistant HSC-1 Cells Show Lower Levels of PpIX and ROS than Parental Cells

**Figure 3:**
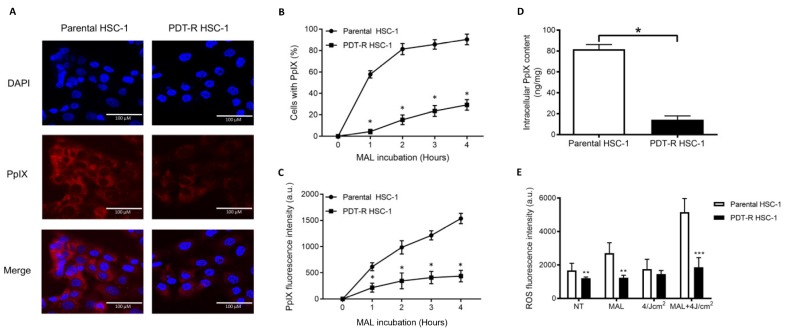
Production of PpIX and ROS in PDT-resistant HSC-1 cells. (**A**) PpIX was found mainly in the cytoplasm of both parental HSC-1 and resistant cells. Nuclei are stained with DAPI (Blue), while PpIX fluorescing in red under blue exciting light (λEx = 460–490 nm). (**B**) Positive cells for PpIX production. (**C**) PpIX fluorescence intensity in parental HSC-1 compared to resistant cells at different times. (**D**) Intracellular content of PpIX measured at λ_Ex_ 406 and λ_Em_ 605 nm using a spectrophotometer. (**E**) Fluorescence intensity of ROS production in parental HSC-1 and resistant cells. Values of *p* < 0.05 were considered statistically significant. a.u. = arbitrary units; * *p* < 0.05; ** *p* < 0.01; *** *p* < 0.001. Data were expressed as mean ± SD of three biological replicates.

To determine the cellular location and intracellular content of PpIX, parental and resistant HSC-1 cells were observed by fluorescence microscopy after incubation with 2 mM of MAL. PpIX was located in the cytoplasm of both parental and resistant HSC-1 cells (Figure 3A). However, parental cells showed 80% positive cells for PpIX at 4 h post-incubation with MAL, while resistant cells showed about 20% (Figure 3B). Also, fluorescence intensity was higher in parental HSC-1 cells than resistant cells (Figure 3C). The intracellular content of PpIX was significantly higher in parental cells (81.81 ± 4.41 ng/mg) than resistant cells (14.24 ± 3.60 ng/mg) (Figure 3D). These findings suggest that parental HSC-1 cells synthesize and accumulate more PpIX than their resistant counterparts.

Regarding the ROS production in parental and resistant cells post-PDT, we found a significant decrease in the production of ROS in resistant HSC-1 compared to parental cells in condition no treatment (NT) or MAL incubation (Figure 3E). In the same way, post-PDT parental cells show significantly higher levels of ROS production than resistant cells (5150 ± 820 a.u. and 1866 ± 570 a.u., respectively). This suggests a pro-oxidant effect of PDT over parental but not over resistant HSC-1 cells. Although a slight increase in ROS levels in resistant HSC-1 was shown, this is not a significant difference compared with the control group (no treatment; Figure 3E).

These results show that PDT-resistant HSC-1 cells are less susceptible to producing PpIX and ROS.

### 2.4. Differential Expression of Genes Involved in Membrane Transport, Metabolism, Cell Stress, Hypoxia and Cell Survival in PDT-Resistant HSC-1 Cells

We evaluated the differential expression of genes associated with a drug-resistant phenotype. These transcripts were placed in three groups: membrane transporters, cell metabolism (enzymes), cell stress, hypoxia, and cell survival. 

**Figure 4:**
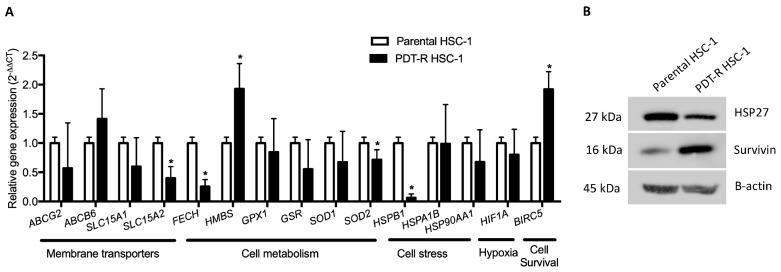
(**A**) Transcriptional expression of genes associated with membrane transporters, cell metabolism (enzymes), cell stress, hypoxia and cell survival in HSC-1-parental and resistant cells. (**B**) Protein expression of HSP27 and survivin in parental and PDT-R HSC-1. Values of *p* < 0.05 were considered statistically significant. * *p* < 0.05. Data were expressed as mean ± SD of three biological replicates.

In the membrane transporter groups, we found a significant downregulation only of *SLC15A2* (PEPT2), a peptide transporter, in PDT-resistant HSC-1 cells (*p* < 0.05). Likewise, in the cell metabolism group both *FECH* and *SOD2* were significantly downregulated in PDT-resistant HSC-1 cells (*p* < 0.05). Meanwhile, only *HMBS* was upregulated in the same cell type (*p* < 0.05). In the cell stress group, we found a significant downregulation of *HSPB1* (HSP27). Also, we found upregulation of *BIRC5* (survivin) in PDT-resistant HSC-1 cells compared to parental cells (*p* < 0.05; Figure 4A). Protein expression of HSP27 (*HSPB1)* and survivin (*BIRC5*) were correlated in parental and PDT-resistant HSC-1 cells (Figure 4B).

### 2.5. Epigallocatechin Gallate Enhances MAL-PDT Efficacy in PDT-Resistant HSC-1 Cells, Improving PpIX and ROS Levels

**Figure 5:**
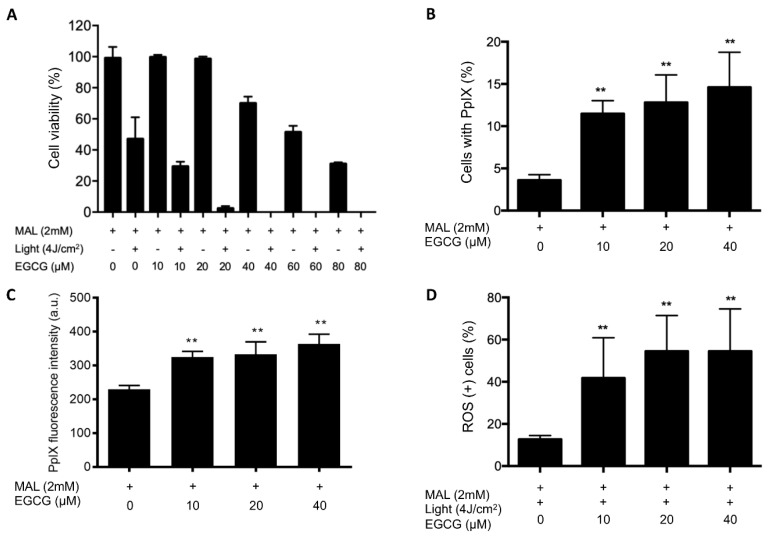
Combined effect of PDT and EGCGT in PDT-resistant HSC-1 cells. (**A**) Cell viability in PDT-resistant HSC-1 cells treated with EGCG. (**B**) Positive cells for PpIX production in PDT-resistant HSC-1 cells treated with MAL and EGCG at different concentrations. (**C**) PpIX fluorescence intensity in PDT-resistant HSC-1 cells treated with MAL and EGCG at different concentrations. (**D**) ROS production in PDT-resistant HSC-1 cells with combined therapy (PDT+EGCG). Each experimental group was compared with their respective control, either MAL (2 mM) or MAL (2 mM) + Light (4 J/cm^2^). Values of P < 0.05 were considered statistically significant. a.u. = arbitrary units; ** *p* < 0.01. Data were expressed as mean ± SD of three biological replicates.

EGCG was used as a potentiator of PDT efficacy in resistant HSC-1 cells. To this end, the cells were treated with MAL and EGCG at different concentrations (10 µM–80 µM). Controls with no light dose were used. In controls without light, low concentrations of EGCG (10 µM–20 µM) had no effect on the PDT-resistant HSC-1 cells. However, as the concentration was increased from 40 to 80 µM, a progressive decrease in cell viability was observed, being statistically significant only at the 80 µM concentration (*p* < 0.05). This shows a cytotoxic effect of EGCG only at high concentrations (Figure 5A). 

Additionally, we found that the cytotoxic effect of PDT + EGCG combination at low concentrations was harmful to the resistant cells. Conventional PDT eliminates approximately 50% of resistant cells, whereas the cytotoxicity of the PDT treatment combined with EGCG increased significantly in all concentrations tested (10–80 μM). It is interesting to note that, at 40 μM EGCG, cell viability decreased to 0% (Figure 5A). These findings suggest an enhancing effect of EGCG on PDT.

Furthermore, to evaluate the effect of EGCG on the levels of PpIX and ROS in resistant cells, 10 μM, 20 μM and 40 μM EGCG concentrations were selected, keeping the concentration of MAL constant (2 mM). Results show that PDT-resistant HSC-1 cells incubated only with MAL (approximately 3.6 ± 0.66% of the population analyzed) were positive for PpIX, with a fluorescence intensity of 229.20 ± 11.50 a.u. By contrast, EGCG in combination with MAL significantly increased the content of PpIX in all concentrations tested (Figure 5B,C). Moreover, PDT combined with EGCG significantly increased the ROS production in PDT-resistant HSC-1 cells. Conventional PDT induces ROS production in approximately 12.71 ± 1.81% of the cells. Meanwhile, PDT combined with EGCG at 10 μM or 20 μM induces ROS production by 41.76 ± 19.11% or 54.53 ± 16.92% of the PDT-resistant HSC-1 cells, respectively (Figure 5D). These results suggest a potential pro-oxidant effect of EGCG in PDT-resistant cells.

## 3. Discussion

Photodynamic therapy (PDT) offers optimal clinical and cosmetic outcomes for NMSC lesions such as basal cell carcinoma or Bowen’s disease and actinic keratosis, with the latter being the precursor lesions of squamous cell carcinoma. In addition, PDT has been proposed as a valid therapy in combination with chemotherapy and radiation therapy or as an alternative procedure in breast and lung cancers resistant to conventional treatments [49,50]. However, previous studies have reported varying response rates for PDT after treatment of NMSC (37% to 100%), which could be explained by the type of lesion and follow-up time reported in each clinical trial [14,15,16,17,18,51].

In our study, we developed a MAL-PDT-resistant cell line of squamous cell carcinoma (HSC-1 cells) to characterize this model and to evaluate the in vitro effect of EGCG as a PDT enhancer.

### 3.1. Characterization of Resistant HSC-1 Cells

A PDT-resistant cell model aids in assessing the efficacy of new photosensitizers, different cell pathways or mechanisms involved in cell survival, etc. In vitro models of PDT resistance have been developed using cell lines from neuroblastoma (SK-N-MC) [52], human colon adenocarcinoma (HT29) [52], human bladder carcinoma (HT1376) [52], human oral cancer (FaDu) [53], human squamous cell carcinoma (SCC-13) [35] and human breast cancer (ABCG2—high MA11) [22], human osteosarcoma cells [54] and murin basal cell carcinoma [55]. Mainly, these protocols use several cycles of PDT, where surviving cells are exposed to a new PDT cycle [35,52,53,55]. Our PDT resistance model has shown to be resistant to death with different light doses in a range of 1–4 J/cm^2^, which can kill parental (sensitive) HSC-1 cells. While, 100% of parental HSC-1 cells were eliminated with a light fluence at 4 J/cm^2^, approximately 55% of PDT-resistant HSC-1 cells survived. Due to the method of selecting resistant cells (a culture of surviving cells after different PDT cycles), a heterogeneous cell population is obtained, which explains why there are cells with different degrees of resistance to PDT.

Interestingly, this resistant phenotype can be demonstrated even two months after cells are frozen.

On the other hand, the cellular response against the cytotoxicity generated by PDT was evaluated by flow cytometry, using Annexin V (AV) and propidium iodide (PI). It was observed that parental cells had higher AV and PI stains than resistant cells, which means that apoptosis signals (translocation of phosphatidyl serine) and cell death signals (positive FS and iodide labeling) had a stronger significant cytotoxic effect on parental cells after 1 h of treatment.

In addition, this resistant model was characterized through the measurement of PpIX by confocal microscopy, flow cytometry and spectrophotometry, demonstrating that resistant HSC-1 cells had lower levels of PpIX than parental HSC-1 cells. These results agree with those found in SCC-13 cells also studied by flow cytometry [35]. The reasons why resistant cells show less content PpIX than parental cells could be due to their ability to be synthesized and accumulated within the cell [56,57]. First, PpIX is synthetized in the heme pathway from precursor MAL, which needs to be converted into ALA before entering the heme synthesis cycle [30,57]. Hydroxymethylbilane synthase (HMBS), also called porphobilinogen deaminase (PBGD), is responsible for the conversion of the porphobilinogen molecule to hydroxymethylamino [56], a precursor of the pyrrolic ring of PpIX. Therefore, decreased gene expression or enzymatic activity of HMBS could be associated with low PpIX production. Another enzyme associated with the PpIX synthesis pathway is ferrochelatase (FECH). FECH inserts Fe^+2^ into the PpIX molecule to produce the heme group. Therefore, an increase in FECH activity/expression or an increase in the concentration of Fe^+2^ would affect the accumulation of PpIX in the mitochondria, since it would favor the formation of the heme group [30,57]. In this work, the relative gene expression of the mRNA coding for these enzymes was evaluated. Analyses of 2^−ΔΔCT^ showed that *HMBS* was overexpressed in resistant cells, whereas *FECH* was downregulated compared to the parental cells. These results disagree with what is expected for resistant cells that show low PpIX content. However, analyses of protein expression and/or enzymatic activity are needed to determine with greater certainty the participation of these and other enzymes in the synthesis and accumulation of PpIX from MAL [29,56,58]. Nevertheless, it is known that HMBS is highly active and upregulated in tumor cells vs. non-tumor cells. This is one of the reasons why PpIX is produced selectively in large quantities by the tumor cells of a tissue and in smaller quantities in normal cells [29,56]. A second phenomenon that affects PpIX levels in the cell corresponds to PpIX efflux after being synthesized by the cell. Transporters such as ABCG2, ABCB6, PEPT1 and PEPT2 participate in this mechanism, decreasing the concentration of PpIX within the cell and modifying the efficiency of PDT, because the efflux directly affects the accumulation of PpIX within the cell [59,60,61]. The only significant difference was found in *SLC15A2* (PEPT2), expressed 0.4 times in the resistant cells compared to the parental cells. It was expected that some genes from this set would be overexpressed, and thus explain an increased PpIX efflux mechanism. However, more detailed studies (transporter activity, protein expression) are needed to determine if PpIX efflux levels may actually be affecting the accumulation of PpIX in these resistant cells.

On the other hand, consistent with low levels of PpIX, the MAL-PDT-resistant cells showed lower ROS levels than the parental cells. Therefore, it is clear from these findings that the resistant cells can survive MAL-PDT, because they do not synthesize/accumulate the necessary amount of PpIX from MAL and, as a consequence, insufficient ROS are generated to cause the irreversible cytotoxic effect or necrotic damage. This stage is the most critical in terms of the effectiveness of the treatment, since it is known that cellular damage is greater and faster when ROS levels are high, as can be seen in the parental HSC-1 cells [62,63]. In the same way, knowing that ROS levels are much higher in the parental cells, it is corroborated that they are responsible for the cell death signals (apoptosis, necrosis) previously described.

Furthermore, the relative gene expression of proteins associated with cell stress (*HSPB1*, *HSPA1B* and *HSP90AA1*), hypoxia (HIF1A), cell survival (*BIRC5*) and antioxidant enzymes (*GPX, GSR, SOD1, SOD2*) was evaluated. Of all these genes, it was found that *HSP21* (HSP27) and *SOD2* genes had a low expression in cells resistant to MAL-PDT, whereas BIRC5 was significantly overexpressed. In addition, protein expression of HSP27 and survivin (BIRC5) were analyzed. The levels of HSP27 and survivin were correlated with their relative gene expression. HSP27 is a protein with different roles. In general, it is a chaperone protein, with antioxidant function, inhibiting apoptosis and participating in the remodeling of the cytoskeleton [64]. However, the PDT-related function of HSP27 is open to discussion. For example, overexpression of HSP27 protected colon cancer cells (CaCo-2) and resistant squamous cell carcinoma cells (SCC-13) from the PDT effect (PS: MAL), modulating autophagy as a survival mechanism, whereas the suppression of the expression of this protein contributed to the inhibition of autophagy and the promotion of apoptosis [65]. Unlike the above, this resistant phenotype of HSC-1 cells showed a low gene expression of HSP27, which is consistent with what has been reported in resistant cells of oral cancer (FaDu), where the deregulation of HSP27 was associated with an increase in autophagy and survival [24]. Therefore, it is important to perform a protein-level analysis to establish the role of HSP27 in these resistant HSC-1 cells associated with studies of autophagy and cell death. On the other hand, the high expression of *BIRC5* is very important, given that the *BIRC5* gene encodes survivin protein, which is widely known for its antiapoptotic effect [66], association with resistance to cancer drug treatment [67], or as has been seen in resistance to PDT [23,35]. Due to the above, survivin has been shown to be an important target in PDT [54,68].

### 3.2. EGCG Enhances MAL-PDT Cytotoxic Effect to Treat Resistant Skin Cancer Squamous Cells

The results showed that HSC-1 cells resistant to MAL-PDT were able to survive PDT with 2 mM MAL and 4 J/cm^2^ of fluence. However, the addition of EGCG, in concentrations of 10–80 μM, to the MAL solution improved the cytotoxic effect of MAL-PDT. The results showed that the viability of the resistant cells decreased significantly to 30%, only by exposing them to 80 μM EGCG + 2 mM MAL without light. This effect is associated with EGCG since it is only an antiproliferative agent [69,70,71], while the combination of PDT (2 mM MAL + light) with EGCG in concentrations of 10 μM to 80 μM allowed the viability of resistant cells to decrease to 30%, 2.5%, and 0%, using the concentrations of EGCG 10 μM, 20 μM, and 40–80 μM, respectively. Therefore, when resistant cells were incubated simultaneously with MAL and EGCG the cytotoxic effect of PDT was better.

In relation to the effect of EGCG on synthesis of PpIX, a significant increase in the value of the fluorescence intensity and the percentage of PpIX positive cells was observed. This increase in PpIX content was not similar to the high content detected in parental cells. However, this slightly significant increase in PpIX could be associated with the chelating action of EGCG. Its action as a chelant contributes to its antioxidant activity because it prevents redox activation by transition metals such as Al^3+^, Cu^2+^, Fe^3+^ and Fe^2+^. The structures responsible for this chelating role of EGCG are the 3,4-dihydroxyl groups of ring B, as well as the chelate group (these groups are capable of joining several metals with strong positive charges) [72]. Therefore, the chelation of Fe^3+^ by EGCG decreases the availability of Fe^2+^, which together with PpIX form the heme group by the action of the ferrochelatase enzyme. As a result, higher PpIX content is accumulated in the cell. Consequently, with the PpIX increase associated with EGCG, a significant increase of ROS production in the cells was observed. Although the main reported property of EGCG is its antioxidant activity [43], it also shows a pro-oxidant effect, as reflected in the results of this work. Although there is evidence that EGCG promotes oxidative stress [72], the mechanism by which it acts on PDT is unclear. It is known that EGCG, after being in contact with components of the cell culture medium, can be oxidized and form radicals of semiquinones, superoxide and H_2_O_2_. However, the generation of H_2_O_2_ mediated by EGCG is lower in the presence of cells, due to the activity of enzymes such as glutathione peroxidase and catalase [72]. In this work, the effect of EGCG + MAL was enhanced with light. In controls of EGCG + MAL without light, no significant effect on cell viability was detected in concentrations ≤ 40 μM of EGCG, as observed in these concentrations when light was used. Therefore, based on these findings, the pro-oxidant effect of EGCG is triggered when there is a burst of ROS. Although it is low, considering the levels of PpIX and ROS of resistant vs. parental cells, it is sufficient for an interaction with functional groups of EGCG molecules. Regarding the effect of EGCG on PDT, it has been seen that in human Jurkat cells (leukemia T lymphocytes), EGCG increases the effect of PDT (PS: phloxine B) through an increase in ROS production, specifically H_2_O_2_, inducing apoptosis (caspase-3 activation) [47]. In addition, EGCG showed a weak effect as a photosensitizer alone [47]. Similarly, EGCG has improved the effect of PDT in the treatment of subcutaneous tumors in mice. EGCG was injected for 20 days, after PDT, in tumors of TC-1 cells in C57BL/6 mice (PS: Radachlorin), causing a significant decrease in tumor volume [48]. Similarly, EGCG was injected for 10 days after PDT in subcutaneous tumors of BA cells in C3H mice (PS: Photofrin). The result was an increase in the efficacy of PDT, an increase in apoptosis and a decrease of survival and angiogenic molecules within the tumors. In addition, 90 days of follow-up after PDT, a 50% resolution rate of tumors was obtained only in those treated with PDT, while PDT combined with EGCG (intratumoral injection after PDT) the response to treatment reached 80% without recurrence of the tumor [46].

## 4. Materials and Methods

### 4.1. Cell Culture

Human skin cell line HSC-1, a derivative of squamous cell carcinoma, was obtained from the JCRB Cell Bank. HSC-1 cells were cultured in DMEM high glucose (Hyclone, Grand island, NY, USA) supplemented with 20% fetal bovine serum and 1% penicillin/streptomycin. Cells were maintained in a humidified incubator at 37 °C with 5% CO_2_.

### 4.2. Photodynamic Therapy

Cells were cultured in 24-well plates (105 cells/well). Twenty-four hours later, cells were washed once with Dulbecco’s phosphate-buffered saline (DPBS) and incubated with methyl aminolevulinate (MAL; PDTPharma/ChemScene, Cravinhos, Brasil) at a concentration of 2 mM diluted in serum-free DMEM without phenol red. Then, cells were incubated in darkness for 4 h in a humidified incubator. Immediately, cells were irradiated with red light (630 nm) and 30 mW, using a light-emitting diode (LED) device. To evaluate the cell sensitivity to PDT, different fluences were used (0, 1, 2, 3, and 4 J/cm^2^). Three controls were prepared: cells incubated with medium (no treatment control), cells incubated only with MAL 2 mM (MAL control), and cells incubated with medium and irradiated (light control). After irradiation, medium was removed and replaced with complete culture medium and cells were kept in a humidified incubator.

### 4.3. Generation and Validation of PDT Resistance Model

The HSC-1 cells were used as a cell model to develop PDT resistance through cycles of PDT with increasing light doses (1–4 J/cm^2^). Cells were seeded in 24-well plates and PDT was carried out as previously described. The treatment conditions with survival rates of 10–15% were chosen. Surviving cells were reseeded until reaching 80–90% confluence, to be then re-exposed to a new PDT cycle. A total of 10 PDT cycles were performed: the first two cycles were carried out using 1 J/cm^2^ of light dose, followed by two cycles of 2 J/cm^2^, two cycles of 3 J/cm^2^ and finally four cycles of 4 J/cm^2^. Resistant cells were frozen at −80 °C and stored for at least two months, and then thawed and reseeded. After three passages, PDT resistance was checked by exposing the reseeded cells into a new protocol (1, 2, 3 and 4 J/cm^2^). Cell viability was again evaluated by the MTT assay 24 h post-PDT. PDT-resistant HSC-1 cells were compared with parental cells (HSC-1 cells with original PDT sensitivity).

### 4.4. MTT Assay

Cell viability was evaluated by MTT (Sigma-Aldrich) 24 h post-PDT. Metabolically active cells reduce MTT (3-[4,5-dimethylthiazol-2-yl]-2,5- diphenyltetrazolium bromide) to insoluble purple formazan dye. MTT solution was prepared in serum-free DMEM medium without phenol red at a final concentration of 0.3 mg/mL. The medium was discarded, and cells were washed once with DPBS 1X. MTT was added and cells were incubated for 2 h in darkness at 37 °C with 5% CO_2_. Then, medium with MTT was removed and formazan crystals were dissolved with isopropanol. Absorbance was measured at 570 nm with a background subtraction at 690 nm using a multi-well plate reader.

### 4.5. Cell Death Assay

Cell death assay was evaluated by flow cytometry using the Dead Cell Apoptosis Kit with Annexin V Alexa Fluor^®^ 488 & Propidium Iodide (PI) (Molecular Probes—lifetechnologies, Eugene, OR, USA). One hour after PDT, the medium was discarded and cells were washed once with DPBS 1X. Next, cells were trypsinized, centrifuged and washed with DPBS 1X. The pellet was resuspended in 100 μL of DPBS 1X, stained with Annexin V Alexa Fluor^®^ 488 and PI and analyzed by a flow cytometer (BD FACSCanto™ II), according to the manufacturer’s instructions.

### 4.6. Cellular Localization of PpIX

PpIX fluorescence was observed in HSC-1-parental and resistant cells using a confocal laser microscopy (Olympus Fluoview 1000, Tokyo, Japan). Cells were seeded into chamber slides (2 × 10^4^ cells/well), and 24 h later were incubated with MAL for 4 h as described above. Then, cells were washed twice with DPBS 1X and stained with DAPI.

### 4.7. Detection of PpIX by Flow Cytometry

Cells were seeded in 24-well plates (10^5^ cells/well) and cultured for 24 h. Next, cells were incubated with a 2 mM solution of MAL for 1, 2, 3, and 4 h as described above. Cells were detached by trypsinization and centrifuged and washed as described above. The pellet was resuspended in 100 μL of DPBS 1X for subsequent analysis in a flow cytometer.

### 4.8. Intracellular Content of PpIX

This method was modified from the method of Lee et al. [73]. Parental HSC-1 and resistant cells were seeded in 12-well plates (2 × 10^5^ cells/well) and cultured for 24 h. Next, cells were incubated with a 2 mM MAL solution for 4 h as described above. Cells were washed twice with DPBS 1X and lysed with a cell scraper. A volume of 300 μL of extraction solution (perchloric acid 1 M: methanol, 1:1) was added to each well and cells were incubated for 10 min in darkness at room temperature. Cell extracts were transferred into 1.5 mL microcentrifuge tubes and centrifuged for 10 min at 1000× *g* at 8 °C. A volume of 100 μL of each supernatant was put into black 96-well plates with a clear bottom. To calculate PpIX concentration, a standard curve was prepared using exogenous PpIX to define a concentration range from 0 to 100 ng/mL of PpIX dissolved in extraction solution. PpIX fluorescence was measured at λ_Ex_ 406 and λ_Em_ 605 nm using a SynergyTM HT multimodal detector (BIOTEK). The PpIX concentration (ng/μL) was associated with protein content (mg/μL) of extracts. Proteins were extracted with RIPA buffer and a cocktail of protease inhibitors. To measure protein content, the BCA Protein Assay kit was used according to the manufacturer’s instructions.

### 4.9. ROS Detection

To detect ROS, a CM-H_2_DCFDA non-fluorescent probe (Molecular Probes—Invitrogen), a general oxidative stress indicator, was used. About 1 × 10^5^ cells were seeded into 24-well plates and, after 24 h were incubated with a 2 mM MAL solution for 4 h. Cells were washed once and incubated with CM-H_2_DCFDA 1 μM for 40 min in darkness at 37 °C. Immediately, cells were irradiated with red light (4 J/cm^2^), except for the MAL control and NT control cells. Then, the medium was discarded and replaced with fresh complete medium to be incubated at 37 °C for 10 min. Cells were trypsinized, centrifuged, and the pellet was washed with 1 mL of DPBS 1X. Then, the CM-H_2_DCFDA signal in cells was analyzed by flow cytometer using λ_Ex_ 492–495 nm and λ_Em_ 517–527 nm.

### 4.10. RNA Extraction and RT-qPCR

RT-qPCR was used to evaluate the differential gene expression of the transporters associated with PpIX efflux (*ABCG2, ABCB6, SLC15A1*, *SLC15A2*), the enzymes involved in heme group synthesis (*HMBS, FECH*), the enzymes with antioxidant activity (*GPX1*, *GSR*, *SOD1*, *SOD2*), cell stress (*HSPB1*, *HSPA1B*, *HSP90AA1*), hypoxia (*HIF1A*), and cell survival protein (BIRC5) between parental and resistant cells. RNA extraction was performed using TRIzol^®^ reagent (Invitrogen). The cDNA was prepared from 1 μg of RNA using M-MLV Reverse Transcriptase (Promega, Madison, WI, USA) following the manufacturer’s instructions. The qPCR was carried out using Brilliant II SYBR^®^ Green QPCR Master Mix with ROX (Agilent Technologies, Cedar Creek, TX, USA). Relative mRNA expression was determined using the 2^−ΔΔCT^ method [74], with *ACTB* and *GAPDH* genes as controls. The primer sequences are detailed in Table 1.

### 4.11. Western Blot

According to relative gene expression results, HSP27 and Survivin (BIRC%) were selected for protein expression analyzes. Parental HSC-1 and resistant cells were lysed using a RIPA buffer (50 mM Tris, pH 7.2; 150 mM NaCl; 1% Triton X-100; and 0.1% SDS) containing protease and phosphatase inhibitor cocktail (1:100, Thermoscientific, Rockford, IL, USA). Protein concentrations were determined by a bicinchoninic acid assay (ThermoFischer, Waltham, MA, USA). Fifty micrograms of proteins were separated by SDS-PAGE on a 12% NuPAGE^®^ Bis-Tris Precast Gel (Invitrogen) and transferred to PVDF membranes (Millipore, Burlington, MA, USA). Protein expressions were detected through the use of rabbit monoclonal antibodies against HSP27 and survivin (1:1000, Cell Signaling Technology, Danvers, MA, USA). All antibodies were diluted in TBST-1% BSA solution (Cell Signaling Technology). The expressions of these proteins were standardized to human β-actin using a rabbit monoclonal anti-β-actin antibody (1:5000, Cell Signaling Technology, USA). Primary antibodies were detected using goat anti-rabbit horseradish peroxidase (HRP)-conjugated secondary antibodies (1:10,000, Santa Cruz Biotechnology, Dallas, TX, USA). Immunoreactive bands were visualized through chemiluminescence in a MyECL image platform (ThermoFischer).

### 4.12. Combined Treatment of EGCG and PDT

To evaluate the effect of EGCG in MAL-PDT-resistant cells, these cells were cultured in 24-well plates (1 × 10^5^ cells/well). After 24 h, cells were incubated with 2 mM MAL and EGCG (10–80 μM) solutions in DMEM without phenol red for 4 h in darkness at 37 °C. The PDT protocol was performed as indicated above using 4 J/cm^2^. Cell viability was evaluated by MTT assay 24 h after PDT. Controls without light were used.

The production of PpIX was evaluated by flow cytometry as indicated above. This time, the cells were incubated with 2 mM MAL, as control, and with 2 mM MAL + EGCG (10, 20 and 40 μM) without irradiation. In addition, to evaluate ROS generation, this time the MUSE^®^ cell analyzer (Merck, Millipore) was used to detect the ROS (+) cells. Cells were incubated for 4 h with MAL (2 mM) and MAL (2 mM) + EGCG (10, 20 and 40 μM) then irradiated with 4 J/cm^2^. Immediately the cells were trypisinized, washed and incubated at 37 °C with the Muse™ Oxidative Stress kit probe (Merck, Millipore) following the manufacturer’s instructions. The samples were analyzed in the MUSE^®^ equipment.

### 4.13. Statistical Analysis

Data were presented as mean ± SD, and significance was analyzed with a Mann-Whitney test using GraphPad Prism (GraphPad Software, La Jolla, CA, USA). All assays were performed using technical and biological triplicates. Statistical significance was established at the *p*-value < 0.05 with a 95% confidence interval.

## 5. Conclusions

The resistant MAL-PDT model of skin cancer squamous cells (HSC-1) is a reliable and useful tool to understand PDT cytotoxicity and cellular response. In this study, we found that PDT-resistant HSC-1 cells were successfully sensitized with epigallocatechin gallate, probably due to their chelating, pro-oxidant, and/or antiproliferative properties. Nevertheless, while further assays are needed to clarify the EGCG mechanism of action in MAL-PDT, the in vitro EGCG effect as an enhancer of MAL-PDT on resistant cells to treat difficult skin cancer lesions is promising.

## 6. Patents

These results are protected by patent application to the World Intellectual Property Organization (WIPO), PCT/IB2019/054042, reference number 2019-17309.

## Figures and Tables

**Table 1:** Primer sequences used for qPCR analysis.

ID	Sequences (5′ → 3′)	PCR Product (pb)
*ABCG2* (forward)	CAGGTGGAGGCAAATCTTCG	209
*ABCG2* (reverse)	AGTTGTTGCAAGCCGAAGAG	
*ABCB6* (forward)	CAACGCCGAGAGTTACGAAG	190
*ABCB6* (reverse)	GTCCCCAACCTGTAGCTTCT	
*SLC15A1* (forward)	ACACCCATGCTCAGAGTTCA	163
*SLC15A1* (reverse)	TACCCATGATGTTGCCCTGT	
*SLC15A2*(forward)	GCAGCTACCACAATATGCCC	173
*SLC15A2* (reverse)	CACTGAACTGTGCCACAACA	
*FECH* (forward)	CCGTATGAGCTCCTGTCGAT	183
*FECH* (reverse)	GGGTTTCAGGTGAGGTGAGA	
*HMBS* (forward)	AGCCTGTTTACCAAGGAGCT	191
*HMBS* (reverse)	GGCAGGGTTTCTAGGGTCTT	
*GPX1* (forward)	CCAGTCGGTGTATGCCTTCT	221
*GPX1* (reverse)	CGTTCTCCTGATGCCCAAAC	
*GSR* (forward)	CAACGAGCTTTACCCCGATG	171
*GSR* (reverse)	TCGTTGCTCCCATCTTCACT	
*SOD1* (forward)	GGAGACTTGGGCAATGTGAC	196
*SOD1* (reverse)	CACAAGCCAAACGACTTCCA	
*SOD2* (forward)	TCCGGTTTTGGGGTATCTGG	152
*SOD2* (reverse)	TGACGTTCAGGTTGTTCACG	
*HSPB1* (forward)	CCAAGTTTCCTCCTCCCTGT	168
*HSPB1* (reverse)	CTTTACTTGGCGGCAGTCTC	
*HSPA1B* (forward)	GATCAACGACGGAGACAAGC	182
*HSPA1B* (reverse)	GCTGCGAGTCGTTGAAGTAG	
*HSP90AA1* (forward)	GGGGAAAGGGGAGTATCTGG	156
*HSP90AA1* (reverse)	TTTTCTGTGCCTACGTGTGC	
*HIF1A* (forward)	ATGTAATGCTCCCCTCACCC	189
*HIF1A* (reverse)	CCTGAATCTGGGGCATGGTA	
*BIRC5* (forward)	GGTTTATTCCCTGGTGCCAC	242
*’BIRC5* (reverse)	ACTTCTCACCTGGTAAGCCC	
*GAPDH* (forward)	TGCACCACCAACTGCTTAGC	87
*GAPDH* (reverse)	GGCATGGACTGTGGTCATGAG	
*ACTB* (forward)	GACAGGATGCAGAAGGAGATTACT	142
*ACTB* (reverse)	TGATCCACATCTGCTGGAAGGT	

## Abbreviations

PDT	Photodynamic therapy
NMSC	Non-melanoma skin cancer
PS	Photosensitizer
MAL	Methyl aminolevulinate
PpIX	Protoporphyrin IX
EGCG	Epigallocatechin gallate catechin
LD	Lethal dose
a.u.	Arbitrary units
